# The Point Bonitation Method and Its Adaptation in Risk Studies: A Case Study in Sri Lanka’s Cities in the Coastal Zone

**DOI:** 10.3390/ijerph18042060

**Published:** 2021-02-20

**Authors:** Dorota Rucińska, Martyna Zagrzejewska

**Affiliations:** Faculty of Geography and Regional Studies, University of Warsaw, 00-927 Warsaw, Poland; martyna0109@onet.pl

**Keywords:** Point Bonitation Method, Sri Lanka’s cities, socio-economic risk reduction

## Abstract

Article proposes using weighting method named the Point Bonitation Method, a popular interdisciplinary method, especially in the tourism and socio-economic geography, for giving optional direction to further researching tsunami risk. This method qualifies and quantifies those factors that lead to natural disasters so that it is possible to make comparisons with their roles in disaster areas. This case study in Sri Lanka shows a specific result that is quantification of vulnerability by regions and can be used and developed locally for disaster risk management and reduction. This paper presents discussion about other possible reasons of high risk in regions.

## 1. Introduction

Investigations performed in recent decades that took disaster risk reduction into consideration—aside from interest in natural hazards—focussed on exposure, vulnerability, and resilience. These investigations then lead us towards putting together some forms of resistance against any natural hazards. At the same time, *The 2015–2030 Sendai Framework for Disaster Risk Reduction* calls upon us to conduct research activities into vulnerable areas, such as any movement from disaster management to disaster risk management [1].

This study is the adaptation of the Point Bonitation Method when it comes to the identification and estimation of the ratio between exposure and resilience as elements of natural disaster risk. Based on the case of the use of land on the coast using the damage observed after the tsunami—these two elements of risk, as well as vulnerability, were tested as a key to understanding local risk mitigation based on social, economic, and environmental factors for sustainable land-use planning.

There are differing definitions of risk that are both functions of natural hazards and vulnerability [2,3,4,5,6]). The exposure is represented by structure, population, and economy (Bohle 2001) [7], as well as covering critical physical and social infrastructures [8]. Exposure and resilience are very important elements of risk [2,3,4,5,6,9]. Resilience is a system concept, and seen as a desirable property of natural and human systems, including cities and coastal zones in the face of a range of potential stresses [10,11].

Coastal ecosystems are highly resilient because of the diversity of their functions and the linkages between these functions [12], which is very important for this study; it is also about their capacity to absorb such shocks. Resilience can be also understood as being the opposite of vulnerability [13].

Physical investigation concerns the physics of tsunamis [14], the timing and westward progress of the waves after the tsunami in 1998 [15], and inundation distance [16]. Natural ecosystems provide protection against extreme weather events and natural disasters [17].

The degradation of mangrove forests is one of coastal problems faced in Sri Lanka [18], along with in the loss of mangrove diversity [19]. The activity of cutting buffer zones on the coast would increase the risk of tsunami damage. There important is the resistance to tsunami impact on infrastructure [20]. By the second half of the 20th century, 50% of the world’s mangrove forests had been destroyed. Current annual loss rates vary from 1 to 20% [21], but this has also been because of the impact of the tsunami [22]. Studies of the Indian Ocean tsunami impact in 2004 on Sri Lanka were the first to be published [23,24,25,26,27,28]. There was an analyses conducted between 1900 and 2016 that informed of dominating research in Indonesia studies [29]. For further development of DRR, there is a need for study as regards all elements of risk. The idea of coastal management in Sri Lanka is not a new or recent event [30,31], and policies and strategies to DRR, these are often not as successful as expected. Systematic utilization of vulnerability information based on factors for development of tsunami risk reduction in the DRM is still limited, e.g., in areas of Galle; there are many studies on assessment methodologies of risk, however, if no use is made of them, for example, at the local level, then development is of little help [32]. One important issue is to link post-disaster reconstruction activities, along with the key factors to either prevent and decision-making by stakeholders [33,34].

Hazard and vulnerability analyses in Sri Lanka—when considering the specific elements of people, economic activity, and infrastructure—has demonstrated that there are regional variations [35]. Although there was a lessening of tsunami inundation on the west coast, there was confirmed damage from Galle up to Kalutara, and further along the shore. Inundation peaks on this stretch of the coast appeared near Paraliya-Telwatte, Akurala, and Balapitiya [36]. In Akurala, there was also damage despite what had been relatively positive pre-tsunami status [22]. On the southwest coast, the tsunami overturned rail carriages near Peraliya, killing over a thousand people; and the southern railway line from Balapitiya to Galle suffered heavy damage near Akurala [36]. One of the proposals as regards mitigating the effects of a potential tsunami is assessing the contribution of a revetment and a coastal railway embankment, in order to enhance the resilience of the coastal villages of Dimuldoowa and Wenamulla along the southwestern coast of Sri Lanka [37]. However, this concept is disputable. In the Philippines, the traditional knowledge of the fishing population indicated that mangroves have a protective buffering function. The forests are important economic in the local human, but they also function as a physical barrier against tidal and ocean influences. Surveys show that the mangroves were in good condition and are able to protect the coast from natural hazards in 24 mangrove lagoons and estuaries in the southwest, south, and southeast zones except that areas that had been converted, for example, to shrimp farms [22].

Sri Lanka is located in the eastern mangroves region, and generally there is a diversity of twenty-seven mangrove species [38]). Research in Southwestern Sri Lanka have shown damage to houses which decreased the further they were located from the sea as well as revealed differences between three vegetation classes present in the area with regards to water level and damage to property [39]. Mangrove trees experienced moderate to severe damage in December 2004, in the Medilla, Wellaodae, Kapuhenwala sites, and the Oruwella harbour area. On the other hand, trees that stood behind a 50–100 m patch of forest showed only minimal damage. The mixed mangrove system in the Walawe River (Wanduruppa area) had a high impact on trees, and further damage has been recorded to mangroves at Negombo (Sri Lanka) [27,40]. Interior mangrove zones and land areas were largely unaffected [22]. Surveys in Sri Lanka in 2005 have shown that mangroves in good condition can protect the coast from natural hazards. The post-tsunami surveys in Sri Lanka in 24 mangrove lagoons and estuaries in zones (the southwest, south, and southeast) show that quality and the types of mangroves offer protection to populated districts in the coast. Moreover, mangroves are resilient and may be restored [22]. Other forests can protect the coast as the callophylum is a deep-rooted and salt-tolerant tree that can provide protection from tsunamis on the west coast of Tumleo Island [15]. Understanding the role of the mangrove forest has led to new courses of action as, e.g., the Mangrove Action Project (MAP, 1992), mapping of mangrove [40] and working partnerships formed in 2000 for mangrove restoration [41,42]. Identification changes based on a visual interpretation of mangrove species [43] opened up new opportunities for the mapping of mangroves using high-resolution optical data (IKONOS-2 in 1999 and QuickBird in 2001) that allowed two mangrove species to be distinguished also in Pambala; interest in the remote-sensing and mapping of mangroves is growing [22,38,44,45]. However, mangrove forest coverage can sometimes be mistaken for wetland vegetation [46]. Similarly to the mangrove forest, destruction of coral reefs at five sites in the south and southwest (Kapparatota-Weligama, Polhena, Unawatuna, Hikkaduwa, and Rumassala), and at one site in the east (Dutch Bay, Trincomalee) were identified. Before the tsunami, Dutch Bay had a coral cover of about 50%, after which there had been severe destruction as extreme mechanical damage. There was very little evidence of litter, debris and sediment from land sources [26]. However, tsunamis only have a minimal impact on those lagoons that show no ecological degradation (e.g., Komari L. Pottuvil L., Bentota Ganga Estuary, and Kaluwamodara), or they are protected because of their distance from the shore and by mangrove forests Rhizophora spp. (Koggala L., Galle-Unawatuna, and Balapitiya Estuary). Lagoon damage was not linked to tsunami wave energy [22] which may suggest potential protection from wave impact. A case study of tsunami impact in seven Special Area Management (SAM) sites in Sri Lanka affected by the tsunami—Negombo, Lunawa, Maduganga, Hikkaduwa, Habaraduwa, Mawella, and Kalametiya—provides an indicative sample of the impact of the tsunami on the coast [26].

The coastal zone of Sri Lanka is described within both living and non-living resources that include a diversity of coastal ecosystems. The first Coastal Zone Management Plan developed in 1990 was revised in 1997 by the Coast Conservation Department, along with the assistance of the Coastal Resources Management Project. With understanding a need for disaster management after Indian Ocean Tsunami of 2004, the Sri Lanka Disaster Management Act (2005). Politics, however, is criticized; if the Coast Conservation Act (1981) had been effectively implemented, the damage from the tsunami could have been much less because of the presence of natural ecosystems in the coastal zone [47]. Scientists need to pay attention to the necessity of instigating disaster mitigation management plans, which consider the topography of each area concerned, as well as putting together a post disaster planning process [48].

Researchers use both stochastic and deterministic methods. Deterministic studies of tracking wave passages of tsunami are still justified, for example, by using an extreme inundation zone during extreme events [49] or to identify the extent of a tsunami inundation zone by means of field measurement and satellite remote sensing [50].

There is an intensive development of fuzzy logic around the world and wide applications. The fuzzy set is understand as a class of objects with a continuum of grades of membership, and a set is characterized by a membership (characteristic) (Zadeh 1965) [51].

An interesting method is the method of clustering mixed data because there is a difference in the two types of data, and the definition of a cluster is based on similarity. This method allows the use of numerical and nominal data (similar to the point mounting method using quantitative and qualitative data). This is an advantage of the method, because limiting the analysis to one group of data reduces its effectiveness [52,53]

The multi-sectoral approach to analysis has its advantages. An example would be the three-sector model lays the groundwork for analyzing policy choices in more complex sectoral settings (a multisector economy) address the relationship between commodity revenues and manufacturing output with a special focus on the role of the agricultural sector. The main result of the multisector analysis is that not diversification, per se, but rather a diversification with the substantial domestic factor or market orientation has the capability to limit the magnitude of deindustrialization [54].

Credit risk is one of the main risks faced by a bank and is generated by the lending activity to clients. Credit risk assessment should be based on a multi-criteria approach, which enables the evaluation of several variables measured at different scales in a unified manner. Therefore, it is emphasized the relevance of both quantitative and qualitative features of applicants using methodology based on mixed data clustering techniques. Cluster analysis is useful in the estimation of credit risk. In this context, clustering algorithms for mixed data provide the approach using evaluation integrating qualitative and quantitative aspects provides a broader analysis of potential creditors, considering various features and gives more detailed specification of the bank customers profile [55].

## 2. Materials and Methods

What the Point Bonitation Method allows us to do is to group features with different characteristics, along with combining qualitative and quantitative data [56,57,58] and assigning given values of features, according to a fixed scale of values [59,60]. Classification consists of the formation of a relative value series by using precisely defined criteria that takes the individual characteristics of the area into account (physic-geographic situations) [56]). The Point Bonitation Method brings together qualitative features to values of points, and those points are summed up by using simple or complex mathematical functions in order to assess the attractiveness of the environment [59,60,61,62]. Like all methods, it has weak points, but opinions are divided: the system of selection criteria, threshold, and number of ranges can be arbitrary [56,57,63], and the opposite, the method allows obtaining an objective, comprehensive picture of the potential of those features that have been examined [64]. The method is widely used in many disciplines for quality assessment: (i) In spatial planning, in assessing the geo-diversity of the environment, in the protection of forests and green areas, for grading land degradation; (ii) in geography, it is a qualitative evaluation of the elements and characteristics of an environmental structure, for example, topography, woods, lakes, the presence of historical monuments, nature conservation, the presence of tourist infrastructure and communication accessibility; and (iii) it aims to assess the attractiveness and usefulness of the area to be surveyed for tourism and recreational purposes [65,66].

The method is the deterministic method and using local historical data, it allows the possibility of qualifying and quantifying disaster factors in order to make comparisons in disaster areas. Based on the review of the literature (above) were selected factors to this case study.

This paper has focused on two kinds of disaster factors: An anthropogenic element of exposure that generated damage in urban areas, and the natural factors that minimized damage and therefore contributing to coastal resilience. The sensitive infrastructure on the coast and the density of population, as well as mitigating elements of the disaster located within the animate and inanimate areas of nature, were analyzed.

There was an assumption that the risk would be reduced if the ratio resilience and copying capacity to vulnerability and exposure were at its highest. However, sustainable land-use planning requires understanding of the scientific work in the DRR by the decision makers in order to balance community growth with any resilience to natural hazards.

In this kind of study, it may be the case that the structure of the area that has been impacted upon by an extreme natural hazard, or the preservation measures undertaken after the impact, has been destroyed during such infrequent extreme events.

The analysis was based on the geographical data of Sri Lanka in the areas that were affected by the tsunami. Five different administrative centers were selected for the analysis in terms of terrain area, population, and location, as well as the differing exposures to the island’s coastline: Batticaloa, Trincomalee—which is located in the eastern part of the island; Galle; Hambantota; Matara, located in the southern part of Sri Lanka; Colombo; Kalutara, located in the western part of the island; Puttalam—located in the northwest of the island; and Jaffna, located in the northern part of the country.

The data used was from the Food and Agriculture Organization of the United Nations in 2020 [67], whereas the data on average heights above sea level at specific cities were taken from 2003 [68] before the tsunami. Anthropogenic data was selected from materials of the FAO in 2020 [67]. However, data on the number of expressways, hotels, banks, schools, and hospitals were collected on the basis of available large-scale maps [69], and coral reef data before the tsunami [70]. In the study data about completely and partially destroyed houses based on earlier investigations of other authors was used.

Based on the literature review, the factors influencing resilience which in this region include natural features regulating (reducing) wave impact was selected: the surfaces of mangroves and lagoons, topography of the terrain (height above sea level), and the quality of coral reefs (which was described in the introduction).

Significant indicators of exposure are those the destruction of which brings about serious and negative economic effects and has a destructive effect on the functioning of local life. The following numbers were used: expressways of transport and economic importance (motorways, expressways, main national roads), hotels, banks, and sensitive infrastructure, such as the number of schools and hospitals.

The number of assigned weight points depended on the intensity of natural (increasing resilience) and anthropogenic (increasing exposure) elements. The assumptions were made: The greater the intensity or density of the phenomenon, the greater the number of assigned valuation points; the fewer elements, the fewer points will be awarded. Natural and anthropogenic factors, as well as the values of weight points, are summarized in tables. Four equal classes were distinguished, considering the greatest and the smallest value.

Natural disaster risk reduction requires the maintenance and improvement of local resilience features using biogenic and abiogenic nature systems, and increasing any natural resilience and decreasing anthropogenic exposure. Therefore, the aim of this study was to test the Point Bonitation Method for DRR and estimating the ratio between exposure and resilience along the coast after a tsunami. In these studies, the calculation (I = N/A) was used when it came to describing the relationships between exposure and resilience along selected coastal regions; when N is the ‘natural’ and A is ‘anthropogenic’ element. Two elements of vulnerability were subjected to analysis: anthropogenic elements (A) (the density of population, along with any sensitive infrastructure on the coast, plus negative impact and increase of risk which describing social-economic exposure); and natural factors (N) located in those areas of nature that are animate and inanimate, and that result in some form of local resilience and so contributing to some form of minimizing damage, thus resulting in a positive impact and a decrease of risk. The number of factors should be the same within the two groups (group A and in group N for the vulnerability system to tsunami hazard); the greater the intensity of the phenomena, the greater the number of weighting points. Resulting is the index, where high value of the index shows a good situation for region and low value of the index shows a poor situation in region.

In the process of analysis, it was necessary to include other methods allowing for an accurate interpretation of the extracts.

For this reason, the data on houses damages completely (C) and partially (P) were compared. Due to the fact that the main generating and neutralizing factors are found in indicator (I), it was assumed that the differences between C and P in regions are due to a greater number of other factors, e.g., neighborhood management and adherence to building rules and, therefore, quality of buildings. Therefore, it was assumed that where there are: (i) More dilapidated buildings (C > P), there were more buildings without meeting quality standards; and (ii) with more partially damaged buildings (P > C), there are more better-built buildings (i.e., built with a better quality than required).

The main limitation in the study was the basic access to Sri Lanka spatial data (publicly available). In addition, the classification and scoring (weighting) methods may influence the results obtained. A weighting method can be applied to mixed data (which is an advantage of the method) but also may, nevertheless, produce a loss of part of information.

## 3. Results

Based on selected regions along the coast, anthropogenic and natural elements were classified and taken for examination: The density of population, fast roads, hotels and schools, hospitals, and banks exposed to damage (Table 1), as well as the high protective abilities of lagoons, mangroves, and the elevation of coral reefs (Table 2).

Next, various qualities were reduced to numerical values.

The test was based on the geographical data of Sri Lanka in the areas that were affected by the tsunami in 2004 [71].

**Figure 1:**
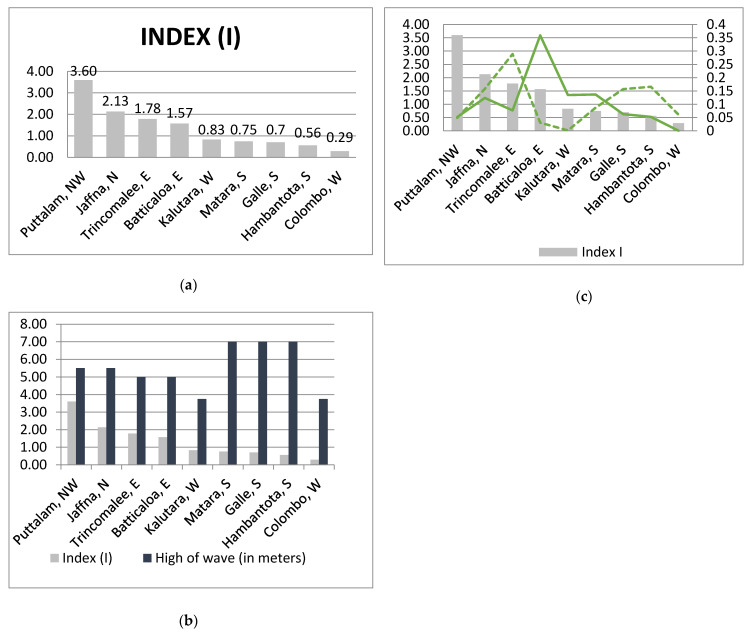
Indeics: (**a**) Relationships between exposure and resilience based on index (I) districts and provinces; (**b**) height of wave and index (I); (**c**) indices of destroyed houses by type: complete destruction and partial destruction of houses.

In the results, the ratio of weighting points has been split into groups that cover districts. Four classes were distinguished based on the grouping of the whole community. The condition for the division was to minimize differences within one class, while maximizing differences between classes. For this purpose, the discontinuity method was used, i.e., characteristic points indicating a breakdown of the set of observations in a sequenced decreasing or increasing order (Figure 1a, decreasing order).

High resilience and low exposure figures were in Puttalam (NW) (I = 3.60). Opposite, high exposure and low resilience was noted in Colombo (West) (I = 0.29).

Based on index (I), regions were divided into 4 groups to better understudy of diversity:

- Very high index, VHI (from 3.001 to 4.0); VHI (3.60) shows very good relationships between the nature and the exposure that is in Puttalam;
- High index, HI (from 2.001 to 3.0); HI (2.13) shows good relationships in Jaffna (2.13) in the north,
- Low index from, LI (1.001–2.0); LI (1.78–1.57): bad relationships in: Trincomalee (1.78) and Batticaloa (1.57) on the east coast of Sri Lanka;
- Very low index, VLI (from 0 to 1.0); VLI (0.83–0.29): very poor relationships in in Kalutara (0.83) in the west, Matara (0.75) in the south, Galle (0.7), Hambantota (0.56) in the south, and the lowest index in Colombo (0.29).

There were extremely different natural-anthropogenic relationships that characterize selected cities, such as in Puttalam (VHI 3.60 and vary good relationships between resilience and exposure) and in Colombo (VLI 0.29 and very bad relationships) (Figure 1a)**.** In three districts: Matara, Galle, and Hambantota located in the south and also in regions of very low index (I) were the higest waves (Figure 1c)

### Complementary Study

The analysis showed that the coincidence of low index I and very high waves (Figure 1b) is not reflected in the damaged houses (Matara, Galle and Hambantota) and the peaks of damaged houses both kinds were in Batticaloe and Trincomale in the east (Figure 1c). The most destroyed houses in total (HCD Index) were in Batticaloa in the east (5 m wave height) and in Trincomalee (5 m wave height) also in the east, where partly-destroyed houses were also the greatest (HPD index) (where indices were equal to the ratio of the number of homes destroyed to all homes destroyed in these regions) (Figure 1c).

Since the main factors generating and neutralizing factors are included in index (I), it was assumed that the differences between C and P in regions are due to more other factors, e.g., how to manage the district and comply with construction rules and, consequently, the quality of buildings. It was assumed that where there are: (i) More completely-destroyed buildings (C > P), where there were more buildings without maintaining quality standards; and (ii) more partially-damaged buildings (P > C), where there are more better constructed buildings (i.e., those of better quality built than is required).

Studying the ratio of the numbers of the kind of house damage, completely (C) and partially (P) (Table 3), shows that there are:

• Four regions with C > P ratio where can direct impact of poor quality of buildings and weakness management in districts: Jaffna (nearly six times more), Batticaloe (about three times more C); and smaller differences Colombo (about 1/3 more C), Hambantot (about 1/3 more C, less than 1000). Among these four regions is also Jaffna with good resilience and exposure (HI) relations, and Batticaole (LI), where the number of completely destroyed houses was the largest among the districts analyzed. these regions are not distinguished by significant unemployment and poverty.

In Hambantota (VLI) the number of destroyed houses was not so large, and the difference between C and P was the smallest in this group (in Hambantota the wave was 7 m) which allows us to say that the management and quality of buildings was not very bad there.

Colombo is characterized by Colombo, Hambantota (sources from 2002) [72]).

• Five regions with P > C ratio where can direct impact of better quality of buildings and management system in districts: Puttalam (over 3 times more P), Trincomalee (about two times more P), Matara (over two times more), Galle (slight difference—smaller than 500). The advantage of P over C in Puttalam overlaps with the VHI class.

In Trincomalee (LI), at the same time, there is the largest number of partially destroyed houses among the analyzed regions in the country; this will put this district in a slightly better position than Batticaloe. Kalutara (about 1/3 more Pthan C), Matara (nearly two times more P) and Galle (the difference is not more than 500) (VLI): in Kalutara (about 1/3 more P) they are characterized by high unemployment (27% and 26%, respectively) and a large number of people living below the poverty line (sources from 2002). Especially, the situation is in Eastern Provicne and in the same group LI are Trincomalee and Batticaloa. It confirms another factor (s) than used in index (I) which describing relationships between resilience and exposure [72]. The exception is Puttalam, where total damage are low numbers with the amount of partial damage to houses. According to this study, only Puttalam is the region where there are optimal resilience and exposure relationships describing by VHI, relation P > C showing good management and quality of houses.

## 4. Discussion

The analysis shows that most of the analyzed regions have poorly or very poorly developed resilience. In addition, some other factors that have not been known in this study strongly affect almost all regions. Jaffna is such a difficult example, where the exposure and resilience index is high (HI), but the total destruction of houses prevails there.

In addition, in the regions studied, the largest destructions of houses took place in Batticaloa and Trincomalee (both locational in Eastern and have the LI), but in Trincomalee partially-destroyed houses dominated (here the LI was slightly better than in Baticaloa). The question arose: What was the reason for such large damages and kind of them at the same time various types of such damages, apart from the exposure and resilience relations, to the LI? According to the authors, only part of the reasons can be seen in the nature.

Although, in the literature that the greatest damage during the 2004 tsunami occurred in the eastern province, whose coast is more or less parallel to the earthquake crack zone and therefore encounters the upcoming tsunami from the Indian Ocean; 65% of destroyed buildings were located there, this does not convince the authors of this article.

This method unexpectedly directed researchers to a new hypothesis.

Noteworthy are the areas of Galle and Matara, where the wave reached far inland, and these are specific regions with a high percentage of the unemployed, like Kalutara (20%), Galle (26%), and Matara (27%), where there is also a large population below the poverty line [72]. Areas of this type often do not meet construction requirements regardless of the region of the world. According to the authors, the reasons for this situation should also be sought in human activities (or lack of activities for DRR), which may then be manifested in the form of poor quality buildings, and thus also poor quality of site management. Many publications highlight the impact of corruption in countries with natural, especially geological, threats [73,74,75,76,77]. Corruption based on data in 2006, ranking 156 (score, 67.5%) [78]. Investigating corruption is extremely difficult and complex. However, the effects of corruption were also feared after the tsunami in the context of humanitarian aid and the non-toxic use of such aid in Indonesia [79,80]. That is why the hypothesis was put forward that in such a Jaffna region the consequences of a disaster result from poor management or corruption and not the exposure and resilience relationship—similarly in Batticaloa, it seems justified. It cannot be ruled out that in other regions (excluding Puttalam), along with improving the exposure and resilience relationship, research on management quality and corruption in the regions should be worked intensively to properly locate investments and controls to get to reduce disaster risk. This work is necessary if have hypothesis that impact of corruption is dominant in confrontation with the nature and exposure. The proposed Point Bonitation Method requires further tests and consideration of new natural factors (such as the kind of mangrove).

Ethics factors also require separate studies, but they may play an equally important role in DRR in Sri Lanka.

This case study presented the results of the Point Bonitation Method. The advantage of this method is the possibility of evaluation using very simple analytical tools, without access to highly-qualified experts and expensive analytical tools, and various types of quantitative and qualitative data. In the future, the vulnerable research should be developed and include also social aspects as u unemployment, poverty, corruption could be included in the index (I), and the kind and quality of mangrove, and location of fishing farms based on high-resolution data.

Corruption testing is very complex and estimators are non-precise. Despite their merits, the underlying estimators have also a limitation, e.g., the available PMG estimators are parametric specifications, which assume that the functional form of the relationship is predetermined what can lead to econometric mis-specification. One of the presented variables is the Political Rights Index [80].

There is possible augmentation of the generic approach by fuzzy sets [51].

## 5. Conclusions

This study is the first to test the adaptation of the scoring method to the problem, seeing the potential of the local application for disaster risk reduction management, and requires continuation and development, taking into account two parameters in particular: The quality of mangroves, city buildings, as well as graphical visualization using appropriate software.

The present inquiry bridges this gap by laying a common ground for the mentioned strands of the literature and by putting forward a novel approach of exposure-resilience relationships in disaster risk analysis. The method allows you to fill in the gaps:

(i) comprehensively applying quantitative and qualitative data for risk assessment, because limiting the analysis to one group of data would reduce its effectiveness;

(ii) insufficient knowledge and striving to balance two particularly important risk elements, which are the natural elasticity of the natural environment, which through natural exposure creates a barrier and creates resilience for the coast, and the anthropogenic man-made exposure on the inland coast.

(iii) recognizing the presence of exposition and resilience in the geographical environment and their role in risk reduction, which is key to improving local risk management and disaster risk reduction. Ultimately, the use of this method and the introduction of changes to the real environment may gradually lead to a balance in the geographical environment;

(iѵ) apart from the possibility of using high-quality technology and the obtained data, it gives a new, easy to implement and inexpensive risk assessment method, based on basic data collected locally, reducing the cost of the assessment (increasing the possibility of performing the method at low cost, which increases the probability of performing such a test by various types of institutions or organizations); the method can complement other evaluations.

The method can be useful, as this enables:

- Easy calculation of one final value that describes several diverse features for each of the groups (for anthropogenic and natural groups), along with being a straightforward independent local study;
- Characterizing roles in coastal vulnerability using exposure and resilience;
- Identifying areas, regions, or units in the country that require significant work in improving disaster risk reduction strategies, and pursuing resistance aims by adapting to the effects of tsunamis and build back better;
- Indicating a direction of local investment for modifications that are helpful for the protection of the region (for example, improved environmental conditions, quality of spatial management);
- The modification of locally applied strategies for better adaptation to tsunami and disaster risk reduction;
- Adding other factors that describe the vulnerability to tsunami.

The study showed that the height of the wave was not the dominant factor affecting the destruction of houses in the analyzed regions of Sri Lanka, which confirms other studies. The worst situation is in Colombo, a region located in the west that requires greater focus. Opposite situation to Colombo has Puttalam district (safe situation). Moreover, there are other strong factors that also impact the costal vulnerability. The authors suggest that this may be because of the kind of spatial management as well as procedures in building code implementation and, finally, in the quality of buildings, especially in Jaffna where the HI describe good relationships between resilience and exposure, and in Batticaloa (LI) where there is a low index but very high completely-destroyed houses. Additionally, there is a very low index in Colombo and Hambantota, and Galee, Matara, and Kalutara are characterized by high unemployment and a large number of people living below the poverty line.

Regions of Sri Lanka located in the west and south require greater focus with investment for sustainable space planning, and DRR is needed in the country to improve exposure and resilience relationships.

## Figures and Tables

**Table 1:** Classification anthropogenic elements. Sources: [67,69].

Density of Population	Highways	Hotels	Hotels	(# ^1^)
<600	<2	<8	<8	1
601–1200	3–5	9–17	9–17	2
1201–1800	6–8	18–26	18–26	3
1801–2400	9–11	27–35	27–35	4
>2401	>12	>36	>36	5

^1^ A marker ‘#’ shows the weighting points; the assumption being that the maximum of weighting points in the group was 20.

**Table 2:** Classification of natural elements. Sources: [67,68].

Lagoon in Hectares	Mangrove in Hectares	Coral Reefs and Their Condition	Elevation in Meters above Sea Level	(# ^1^)
<5000	<500	Lack	<8	1
5001–10,000	501–1000	Small degradation	9–17	2
10,001–15,000	1001–1500	Degradation	18–26	3
15,001–20,000	1501−2000	Partly degradation	27–35	4
>20,000	>2000	Very good condition	>35^2^	5

^1^ A marker ‘#’ shows the weighting points; the assumption being that the maximum of weighting points in the group was 20.

**Table 3:** Complete and partially house damage (testing) [72].

Districts/Provinces	Index I Group	Completely	Partially	Completely/Partially Damaged Houses
Puttalam, NW	VHI	23	72	P > C
Jaffna, N	HI	6084	1114	C > P
Trincomalee, E	LI	5974	10394	P > C
Batticaloa, E	LI	15939	5665	C > P
Kalutara, W	VLI	2780	3116	P > C
Matara, S	VLI	2362	5659	P > C
Galle, S	VLI	5525	5966	P > C
Hambantota, S	VLI	2303	1744	C > P
Colombo, W	VLI	3398	2210	C > P

## Data Availability

Publicly available datasets were analyzed in this study.
